# Gangrene of the Leg Following Pneumonia

**Published:** 1903-10-24

**Authors:** 


### GANGRENE OF THE LEG FOLLOWING PNEUMONIA.

Dr. C. L. Gibson [1] reports three cases in which gangrene of the leg developed suddenly in the course of acute lobar pneumonia. In each instance the clinical picture was similar and Dr. Gibson thinks it may be assumed that there was a direct connection between the pneumonia and the gangrene. His explanation of the phenomenon is that a coagulum from the pulmonary veins draining the affected area was dislodged, and came to rest at a site—presumably the bifurcation of the popliteal artery—where it caused permanent arrest of the blood supply. In the first case, that of a man aged 67 years, there were signs of acute right lobar pneumonia. On the twelfth day of the illness his temperature dropped from 103·4° F. to 100·2° F., and to 99° F. the next day. On the thirteenth day of the illness he complained of severe pain in the right leg. Two days afterwards the right foot and leg were painful and tender and cold, and the foot was a purplish colour. No pulsation could be felt in the arteries of the foot and leg. Subsequently the foot and lower part of the leg became gangrenous, a line of demarcation forming at the junction of the lower and middle thirds of the leg.

The second instance occurred in a patient aged 43 years. In this case the temperature fell on the seventh day of the illness, and on the same day the patient felt a sudden numbness in the left foot from the ankle down. He soon lost sensation over this area which became cold but did not swell. Two days later severe pain was felt in the popliteal space, and in the leg. Subsequently gangrene developed, and the leg was amputated. The third case was very similar to the other two. On the fourteenth day after the onset of pneumonia, his temperature having fallen to about the normal, the patient, a man aged 61 years, had a sudden attack of intense pain in the left leg, and it was found that he had no sensation in the toes. Two days later there was discoloration extending up to the knee. Amputation was performed through the thigh.

In all three cases the symptoms of vascular obstruction developed suddenly at or about the time of defervescence. None of the cases showed any obvious kidney or cardiac affection, nor did the urine contain sugar. Dr. Gibson believes that the gangrene in these cases was directly attributable to the pneumonia, and not to some other intercurrent process, for the following reasons :—1. The gangrene was of the dry form due to blocking of an artery. 2. The typically sudden onset of symptoms was in favour of embolism rather than thrombosis beginning in the arteries of the leg. 3. The patients showed no obvious vascular and cardiac lesions favouring the theory of the transferring to the leg of an embolism originating in such process. 4. Thrombosis of pulmonary veins in pneumonia has been affirmed by Welch. Against the view that the embolism was due to the detachment of a cardiac thrombus, Dr. Gibson urges the fact that the cases described were not ones of much severity and were not accompanied by signs of severe heart strain. With regard to treatment in these cases, Dr. Gibson is in favour of performing amputation without waiting for the appearance of a line of demarcation provided that the pulmonary condition has sufficiently resolved to allow of the operation being undertaken. Even if a line of demarcation forms in the mid-leg his experience is that it will be advisable to amputate through the thigh.

[1] Annals of Surgery, September, 1903.

